# Measurements of Soil Carbon Dioxide Emissions from Two Maize Agroecosystems at Harvest under Different Tillage Conditions

**DOI:** 10.1155/2014/141345

**Published:** 2014-10-28

**Authors:** Gerosa Giacomo, Finco Angelo, Boschetti Fabio, Brenna Stefano, Marzuoli Riccardo

**Affiliations:** ^1^Department of Mathematics & Physics, Catholic University, via dei Musei 41, 25121 Brescia, Italy; ^2^ERSAF, Regional Agency for Services to the Agriculture and Forests of the Lombardy Region, via Pola 12, 20124 Milan, Italy

## Abstract

In this study a comparison of the soil CO_2_ fluxes emitted from two maize (*Zea mays* L.) fields with the same soil type was performed. Each field was treated with a different tillage technique: conventional tillage (30 cm depth ploughing) and no-tillage. Measurements were performed in the Po Valley (Italy) from September to October 2012, covering both pre- and postharvesting conditions, by means of two identical systems based on automatic static soil chambers. Main results show that no-tillage technique caused higher CO_2_ emissions than conventional tillage (on average 2.78 and 0.79 *μ*mol CO_2_ m^−2^ s^−1^, resp.). This result is likely due to decomposition of the organic litter left on the ground of the no-tillage site and thus to an increased microbial and invertebrate respiration. On the other hand, fuel consumption of conventional tillage technique is greater than no-tillage consumptions. For these reasons this result cannot be taken as general. More investigations are needed to take into account all the emissions related to the field management cycle.

## 1. Introduction

Soils represent the main terrestrial reservoir of organic carbon (OC), containing almost three times more carbon than plants biomass [1].

The release of carbon dioxide (CO_2_) by soil respiration processes, mainly related to the degradation of organic matter in soils, represents the second largest component of the global carbon cycle [2, 3] and can play a relevant role in the climate change.

Bond-Lamberty and Thomson estimated in 98 PgC year-1 the current global emission flux from soil over the Earth's land surface, which is 10 times higher than the total anthropogenic emission fluxes from fuel combustion [4].

Depending on the agricultural practices of soil management, soils may be important sources or sinks of atmospheric carbon, with consequent implications and effects on a global scale [5–7].

Tillage, even if it incorporates crop residues, is considered a practice that favours CO_2_ emissions from arable lands since it improves the ventilation of the top-soils inducing a fast biological oxidation of the organic matter [8–10].

Conservative agriculture practices such as the no-tillage and the minimum tillage techniques, on the contrary, are considered less carbon emissive [11–14], because the mechanical agitation of the soil is avoided or minimized. The new season crop is sown over the previous crop residues in thin furrows, without ploughing. The decomposition of the crop residues lead to an increase of the organic carbon content on the top layer of the soil, favouring its aggregation and stability. However, as observed by Six et al. [15], in a five-year experiment of no-tillage practice in arid climates, the carbon content of the deeper layers may decrease, accelerating the decomposition in the surface layer [16].

The exchange of CO_2_ between arable soils and atmosphere is only one of the aspects of the complexity of the carbon budget of an agroecosystem.

Nevertheless, accurate measurements of CO_2_ effluxes are required to assess whether a crop management technique is better than another in reducing the CO_2_ emission from soils.

Several approaches have been used to assess the exchange of CO_2_ between the ecosystem and the atmosphere [17–19], each of them with their own advantages and disadvantages [20, 21]. The direct methods include the use of static enclosures (“chambers”) on the soil surface [22, 23] and the analysis of the rate of the CO_2_ accumulation (or removal) in the chamber headspace over a certain period of time after enclosing the surface.

The static chamber technique is relatively cheap and easy, and the automation of the process allows the system to perform continuous measurements of soil respiration. On the other hand both static and dynamic chambers methods were criticized for altering soil environment and air-soil pressure gradient thus affecting the fluxes [24] and for removing the turbulent fluctuations [25, 26]. Many different sources of error are possible when chambers are used [27] and underestimations of soil respiration may happen [28] when the rigid collar over which the chambers are mounted is inserted in the soil so deeply that it cuts roots. However different solutions based on proper chamber designs, data analyses, and spatial and temporal sampling regimes were presented for correcting most of these problems [29, 30].

This work was developed in the context of the AGRICO_2_LTURA Project of the Lombardy Region, which was aimed at investigating CO_2_ fluxes from cultivated soil in an intensive agriculture area of the Po Plain (Italy).

Soil CO_2_ fluxes measurements taken in two maize fields managed with different tillage techniques are presented to provide parameters for the soil-atmosphere CO_2_ exchange modeling.

In particular, measurements of CO_2_ fluxes after harvest and for the following two months were analysed evaluating their mean daily patterns and the influence of different environmental factors such as, litter presence, microbial biomass, and wind turbulence. However, it is important to underline that this paper is not intended to provide the carbon budget of the whole crop cycle in the two agroecosystems, but it is referred to a comparison of measurements made only for a short period of time (two months) around harvest.

## 2. Materials and Methods

### 2.1. Sites Description and Agronomical Management

This study was conducted in the agricultural district of Lodi, Italy, located at the so called “basal level of the plain”, just north of the Po river in the Po Valley (Italy).

Two farms located 5 km apart were selected, one located in the San Martino in Strada village (no-Tillage agriculture site, NT site from now on, 45°16′45.53′′N, 9°32′59.62′′E, 69 m a.s.l.) and the other in the Secugnago village (conventional tillage agriculture site, CT site from now on, 45°14′24.68′′N, 9°35′20.64′′E, 66 m a.s.l.). The dominant cropping system for both farms consisted of a cereal forage rotation, which is typical for the region due to its extensive livestock production.

In each farm a maize field was chosen for the measurements.

The soils of the two fields were both Hapli-Cutanic Luvisols (IUSS-WRB, 2007) with a coarse texture, a clay content of 10–14%, and a pH of 5.5–5.9 [31].

The two maize fields were managed with a wheat-maize rotation and were subjected to different ploughing treatments.

At the NT site, as was the case for the previous ten years of management, a no-tillage agriculture practice was employed. Maize seeds were directly planted into the soil covered by wheat residues and weeds, and two herbicide treatments were performed after maize sowing. N and P fertilizations were performed with chemical fertilizers at the sowing and at the emergence of the 6th-7th leaf.

The field at the CT site experienced a conventional 30 cm depth ploughing followed by a harrow clods reduction before the sowing. Then, two herbicide treatments were performed. Fertilization was made with cattle manure before ploughing and with chemical P fertilizer at sowing.

Irrigation in both fields was performed with flooding until soils reached their field capacity.

After the maize harvest, which occurred at the end of August at the NT site and at the beginning of September at CT site, the maize stalks were left on the soils in both sites.

Detailed information on crop management, including fertilization treatments, irrigations, herbicide applications, mechanical treatments, harvest and relative dates can be found in Table 1.

### 2.2. Soil Measurements and Characteristics

Soil characteristics of the selected fields were assessed on February 2012 [32] with the Area Frame Randomised Soil Sampling methodology (AFRSS) described by Stolbovoy et al. [33]. In each site one 20 m × 20 m sampling area was randomly selected, and two additional areas were identified at 80 and 40 m, respectively, from the first one, following an “L” shape disposition. In each of these three areas, soil and litter samples were taken from 9 sampling points dislocated over the whole surface with a cross shape design (for detail refer to [33]). In each sampling point the litter was collected from a surface of 0.63 m^2^ and the soil samples were taken at three different depths: 0–10, 10–20, and 20–30 cm.

The 9 samples were then merged and mixed into a single sample per area, and the three mixed samples of each field were then sent to the lab for the analysis.

The organic carbon (OC) in each soil and litter sample was assessed by means of the Walkley-Black method [34] which consists of the oxidation of carbon with acidic dichromate (Cr_2_O_7_^2−^) followed by the titration of the excess of dichromate with ferrous sulfate. The OC is calculated from the difference between the total dichromate added and the amount of dichromate left unreacted after OC oxidation.

The organic carbon (OC) in the microbial biomass was assessed by the chloroform fumigation extraction technique (CFE) described by Vance et al. [35] and implemented by Tate et al. [36]. Details can be found in the cited references.

The two sites presented very similar OC content of the topsoils (0–30 cm), not statistically different between them: 9.5 gC/kg at NT site and 10.3 gC/kg at CT site, on average. As a consequence, the total carbon stocks (defined as the total amount of the OC stored in the first 30 cm of soil depth) were comparable at two sites: 44.8 t/ha at NT site and 48.7 t/ha at CT site.

The organic carbon of the crop residues in the NT site litter was 4.8 t/ha, an amount corresponding to 10.7% of the total carbon stock in the top-soil (0–30 cm depth).  Table 2 summaries the OC content in the soil, the litter (where present), and in the microbial biomass of the two sites.

### 2.3. Flux Measurement Technique

A system of three automatic static chambers (SASSFLUX,* Ecometrics srl*, Italy) was deployed at the NT site from the middle of July (with green maize) to the end of October 2012, and a second system with four chambers was deployed at the CT site from the beginning of September (with senescent maize) to the end of the measuring campaign (October 2012).

Each system consisted of a central unit where an infrared gas analyzer (IRGA), a pump, 8 solenoid valves and the controlling system were located, and up to 4 measuring chambers deployed to a distance of 5 m from the central unit.

**Figure 1:**
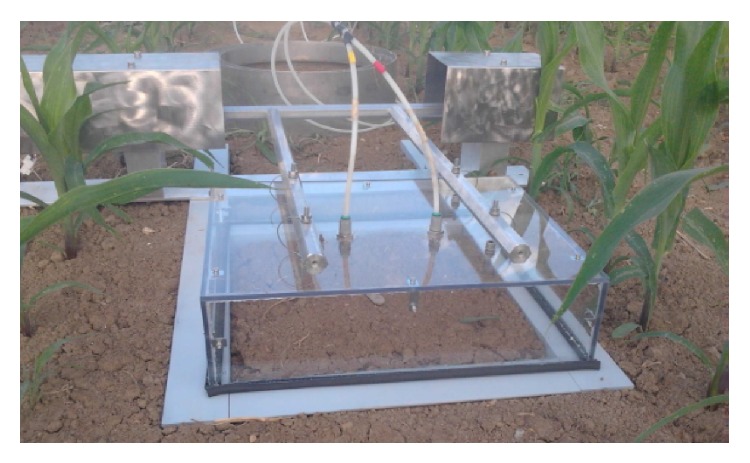
A chamber of the SASSFLUX system.

The measuring chambers (Figure 1) were made of a box-shaped lid of transparent Plexiglas (dimensions 40 × 40 × 10 cm) which is mounted on a steel frame (collar) that is inserted into the soil at a depth of 8 cm, delimiting a measuring soil surface of 35 × 35 cm^2^. The total air volume trapped by each chamber was 16.5 litres taking into account the thickness of the seals.

The chambers were placed below the maize canopy between the rows at a distance of 5 m from the SASSFLUX central unit heading toward the 4 cardinal directions (N excluded at the NT site) and then left until the harvest and the following postharvest period. Measurements restarted one week after the harvest at the NT site and 3 days after the harvest at the CT site. The chambers sequentially closed over their metal collars and the air within was sampled by a 10 L min^−1^ membrane pump trough a Teflon tube (4 mm inner diameter) to the IRGA (Carbocap 343,* Vaisala, SF*) which provided one CO_2_ concentration measurement every second until the chamber lid opened and the soil surface was reexposed to the atmosphere.

To avoid pressure alterations inside the chambers, the sampled air was redirected to the chamber through a second teflon tube after the IRGA analysis.

A personal computer controlled the solenoid valves system which allowed the IRGA to cyclically analyse the air from the different chambers.

The tubing flushing time before and after each chamber closure, and the duration of each measurement session, was set in order to get 4 cycles of measurement every hour (i.e., each chamber was closed 4 times per hour and the closure time was about 3.5 minutes per cycle, including flushing, lid opening/closure and settling time).

The very short closure time allowed the soil disturbance to be kept as low as possible and to gather a high number of measurement replicates in order to get the hourly variation of CO_2_ fluxes.

A very similar system is also described by Jassal et al. [23].

The two SASSFLUX systems were calibrated in laboratory before their deployment in the fields and their instrumental drift was checked at the end of the campaign by performing a one day period of cross-measurements in the same field at the end of the measuring campaign.

The proper operation of the IRGA and the pneumatic system during the field campaign was also checked daily by comparing the daily courses of the soil level atmospheric CO_2_ concentration measured by the two SASSFLUX devices in the two sites.

All of these checks did not reveal any significant difference between the two systems.

Moreover, the SASSFLUX system proved to provide comparable data with other measuring techniques such as vials sampling from the headspace of fixed collars inserted into the soil and subsequent gas-chromatography analysis or sampling made in the same collars with a Brüel-Kjiaer photoacoustical device [37].

### 2.4. Soil CO_2_ Flux Calculation and Data Analysis

The instantaneous flux density of CO_2_ from the soil in each closed chamber, Φ_CO_2__(*t*), is the quantity of CO_2_ that, flowing through the soil surface *A*, is able to increase the CO_2_ concentration of the trapped air volume *V* of a quantity equal to *d*[CO_2_] in the time unit *dt* [22]:

(1) $$\begin{array}{c} \Phi_{{\text{CO}}_{2}}\left(t\right)=\frac{d\left[{\text{CO}}_{2}\right]}{dt}\frac{V}{A}. \end{array}$$

If, as in our case, the CO_2_ concentration is measured as mixing ratio (ppm), the addition of the factor term *P*/(*RT*), where *P* and *T* are the atmospheric pressure and temperature (Pa and *K*), and *R* is the universal gas constant (J mol^−1^ K^−1^), can be used to convert the CO_2_ concentrations to mass density (*μ*mol m^−3^), and the expression of the flux as *μ*mol m^−2^ s^−1^:

(2) $$\begin{array}{c} {\Phi_{{\text{CO}}_{2}}(t)}_{\_\mu \text{mol}\_{\text{m}}^{-2}\_{\text{s}}^{-1}}=\frac{d\left[{\text{CO}}_{2\_\text{ppm}}\right]}{dt}\frac{V}{A}\frac{P}{RT}. \end{array}$$

The flux of the undisturbed soil can be derived by estimating Φ_CO_2__(0), that is, the CO_2_ flux just after the closure of the cambers.

In this work Φ_CO_2__(0) was calculated by linear interpolation of the CO_2_ concentration values versus time during the first 70 seconds of sampling after the chamber's closure in order to get the CO_2_ accumulation velocity (*d*[CO_2_]/*dt*) from the slope of the interpolation line. The selected time window was chosen to ensure the linearity of the first part of the CO_2_ accumulation curve.

In our conditions this simple linear fit guaranteed excellent results even in the (worst) cases of very intense CO_2_ emission rates, when soil is treated with a significant amount of manure.

Two quality controls were employed to check the ill-conditioned samples. The first one regarded the reaching of the full scale value of the IRGA, set to 1000 ppm. When the last CO_2_ concentration measured during the 70 seconds time window exceeded 999 ppm, the sample was considered “saturated” and then discarded. The second one regarded the linearity of the CO_2_ accumulation curve in the chosen time window. When the slope of the interpolation line of the first 70 data was not statistically significant (*F* test with *n* − 2 = 68 degrees of freedom resulting in a probability *P* > 0.01), the sample was rejected. Significant deviation from linearity generally occurred when the lid failed to close tightly, typically when some plant material fell over the collar seals during the chamber closure.

The Φ_CO_2__(0) of the samples which passed the quality check were stored in a common database for the following analysis. For comparison purposes, the arithmetical average of the Φ_CO_2__(0) obtained by all the chambers of each SASSFLUX system in each site (hereafter called CO_2_ fluxes for simplicity) was calculated for selected time periods.

With the aim of comparing the diurnal emission patterns of the two sites, all the CO_2_ fluxes detected by all the SASSFLUX chambers (three for NT site and four for CT site) in the same hour of different days were averaged for a given period in each site. Whenever fluxes were averaged, the standard deviation (*σ*) of the averaged values was calculated and presented. When the averaged fluxes were intended to be meant as an estimation of the real (unknown) fluxes of each hour of the day (and not simply to describe the features of the samples), the associated standard error of the mean was calculated (=$\sigma /\sqrt{n}$, with *n* the number of data used for the calculation of the mean) and presented. The statistical significance of the difference between the daily courses of the CO_2_ fluxes in different periods was tested by means of a one-way ANOVA repeated 24 times, one per each hour, on all the available CO_2_ flux data (all the cambers, all the available data per each hour) grouped by the different periods (the ANOVA factor). Differences with a probability *P* greater than 0.05 were considered not significant.

### 2.5. Data Capturing

Several interruptions in data sampling, due to technical problems or field care needs, occurred during the measuring campaign. The total data capturing, that is, the ratio between the number of the collected samples and the maximum obtainable with a continuously operative system, resulted 88% in the NT site and 91% in the CT site. However, the samples, which passed the two adopted quality control criteria, were 74% and 66% at NT site, and CT site, respectively.

### 2.6. Meteorological Measurements and Weather Conditions

Ancillary measurements of air temperature and humidity (HD9008 DeltaOhm, UK) above and below the crop canopy, barometric pressure (PTB101b Vaisala, Finland), ground heat fluxes (HFP01SC, Hukseflux, Netherlands), and soil moisture (CS616 Campbell Sci., USA; EC5 Decagon, USA) were also monitored at the NT site every 30 s and stored as 15 minutes mean with a CR1000 datalogger (Campbell Sci., USA).

The atmospheric turbulence in the two was measured by means of two colocated eddy covariance measuring stations equipped with a sonic anemometer (USA-1, Metek, Germany) mounted on a mast at 3 m above the ground level.

The turbulence parameter *u* ^*^ (friction velocity) was calculated from the covariance of the vertical and the horizontal wind speed, by following the procedure reported in Gerosa et al. 2003 [38].

**Figure 2:**
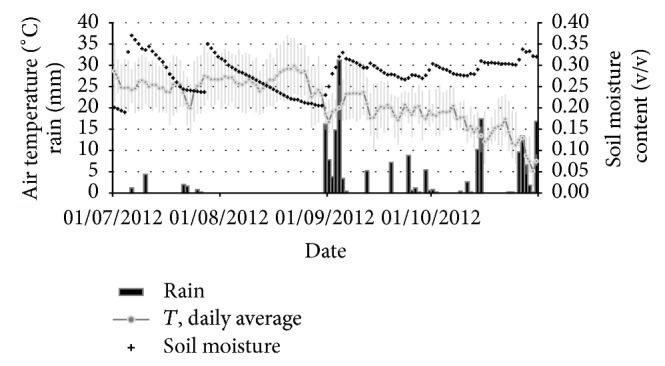
Meteorological conditions at the NT site. Data shown are daily averages of air temperature (°C), total daily rain (mm), and average soil moisture content (volumetric ratio: m^3^ of water per m^3^ of soil). The grayed area around the temperature line indicates the daily maxima and min temperature.

The weather conditions of the measuring campaign are presented in Figure 2 and summarised in Table 3. July and August were sunny and hot with rare strong precipitation events, while September and October were rainy, with quite variable weather in September and more foggy weather in October. The volumetric soil water content of the first 30 cm of soil ranged between 0.19 and 0.37 m_water_^3^ m_soil_^−3^ in the drier months of July and August as a result of the irrigation practice, while in the following September and October months the soil moisture remained quite stable between 0.27 and 0.34 m_water_^3^ m_soil_^−3^. The average soil moisture of the two sites during the common measuring period (September-October) was very similar: 0.294 m_water_^3^ m_soil_^−3^ at the NT site and 0.271 m_water_^3^ m_soil_^−3^ at the CT site.

During July-August the soils were in thermal equilibrium with the below canopy atmosphere (Table 3), while they lost energy in September-October following the removal of the canopy with the harvests and the air cooling.

## 3. Results and Discussion

### 3.1. Soil CO_2_ Fluxes

**Figure 3:**
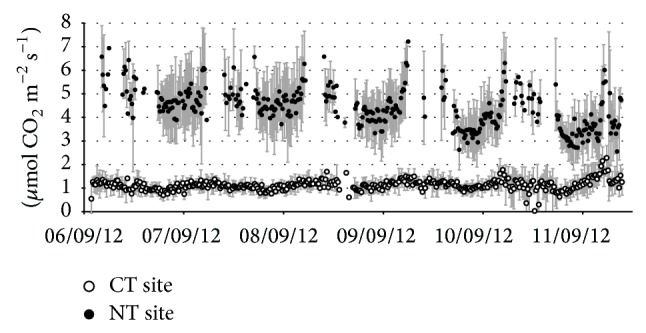
Six consecutive days of soil CO_2_ flux measurements in the two experimental sites. Each point represents the average of the fluxes measured by all the chambers of the SASSFLUX system at each site (3 chambers at NT site and 4 chambers at CT site) in a 15 min period. Vertical bars indicate the standard deviation of the chambers measurements in each 15 min period (CT = conventional tillage site; NT = no-tillage site). Points without vertical bars refer to 15 min periods in which only one chamber was running.

Figure 3 shows an example of one week of measurements at both sites in September. The measurement gaps were due to IRGA saturation conditions, particularly frequent at the NT site during the night.

The NT site experienced higher CO_2_ emission fluxes than the CT site. Around 4 *μ*mol CO_2_ m^−2^ s^−1^ with peaks of 7 *μ*mol m^−2^ s^−1^ and minimum of 2-3 *μ*mol m^−2^ s^−1^ were recorded at the NT site, and about 1 *μ*mol CO_2_ m^−2^ s^−1^ with peaks of 2 *μ*mol m^−2^ s^−1^ were recorded at the CT site.

Moreover the NT site showed a higher flux variability from chamber to chamber, reflecting a greater spatial variability of the organic matter at the microscale. Conversely, the CO_2_ fluxes at the CT site were quite homogeneous, as an expected result of the top soil homogenisation due to the mechanical treatments (see Table 1).

**Figure 4:**
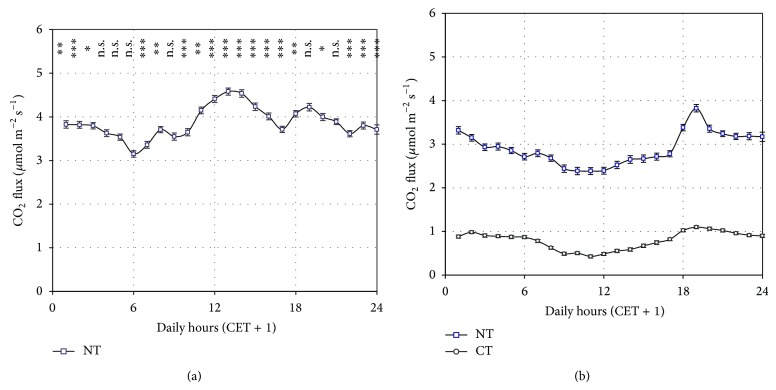
Average daily course of CO_2_ fluxes for the July-August (a) and September-October (b) periods. Each point represents the average of the fluxes measured by all the chambers of the SASSFLUX system at each site for all the days of each two-month periods during the hour indicated in the *x*-axis. Vertical bars indicate the standard errors of the average value. CET + 1 indicate the “legal” time of the Central Europe Time zone. The statistical significance of the differences between the flux daily courses of the two periods in the NT site is reported (for each hour) at the top of the graph (a) (^***^ *P* < 0.001; ^**^ *P* < 0.01; ^*^ *P* < 0.05, and n.s. *P* > 0.05). Differences between the flux daily courses of the NT and CT sites were always statistically significant (^***^ *P* < 0.001) and thus it was not indicated in the graph (b). The maize harvest occurred in both fields between the two periods represented.

Taking into account only the concomitant measuring time period of both sites (September-October), the average flux at NT site was 2.78 *μ*mol CO_2_ m^−2^ s^−1^ while at CT site was 0.79 *μ*mol m^−2^ s^−1^, 3.5 times lower. This difference is also evidenced by the average daily courses of CO_2_ fluxes of the two sites (Figure 4(b)), which resulted significantly different (*P* < 0.01) for every hour of the day.

The CO_2_ fluxes were much more intense in the summer months (June-July) than in the following fall months (Figure 4(a), only comparison on NT site was possible) when they decreased by 42% on average. This was surely caused by the general air cooling, but was also the result of the increased soil exposure to the air after the harvest.

The greater flux variability at the NT site is confirmed by the higher standard deviation of the mean flux estimation for each hour of the day: on average 1.32 *μ*mol m^−2^ s^−1^ in the NT site and 0.50 *μ*mol m^−2^ s^−1^ in the CT site in the common period (Figure 4(b)).

The higher CO_2_ emission fluxes registered at the NT site compared to the CT site was an unexpected result, since the application of no-tillage agriculture practices should have lowered carbon dioxide emissions at the NT site.

This result does not exclude that high CO_2_ losses might have occurred at CT site just after the tillage, as observed by other studies [8]. However, since our measurements were made long after the ploughing treatment (4 months later), they might have missed this initial emission peak.

### 3.2. Potential Role of the Litter

The increase in CO_2_ emissions detected at the NT site may find an explanation if we take into account the presence of a thick organic litter (2.02 ± 0.4 kg/m^2^, Table 2) that could have caused a higher decomposition activity than the one experienced at the CT site, and a different distribution of the organic carbon content in the profile of the first 30 cm of soil between the two sites.

The presence of an organic litter in the NT site, completely absent in the CT site, is the direct consequence of the application of the no-tillage agriculture technique in the past 10 years. Residues of the previous crops were left on the field even during the following crop growing season and never buried by ploughing.

The stimulation of the decomposition activity due to the presence of litter has been well described by Pengthamkeerati et al. [39], Iqbal et al. [40], Li et al. [41], Oorts et al. [42], and Maraseni and Cockfield [43]. For example Pengthamkeerati et al. [39] found that claypan soils with the application of a poultry litter, in an incubation experiment, emitted about the double amount of CO_2_ than the same soils without litter. Li et al. [41], for example, found that the cumulative carbon emissions from mollisols amended with maize crop residues applied on the soil surface was up to 4 times greater than the not-amended soils. The field trials of Pengthamkeerati et al. [39] confirmed their lab findings, but a great interannual variability was highlighted. These results are well in agreement with those found by De Neve and Hofman [44] with crop residues used as an organic amendment.

Morever the stimulation of CO_2_ emission due to crop residues is independent on the nitrogen addition, as Iqbal et al. [40] found in red (Ultisol) soils after the application of straws and different amounts of nitrogen fertilisers.

### 3.3. Soil Organic Carbon Profile and Microbial Biomass

The OC content of the topsoil in the two sites was comparable (Table 2), but the OC content in the surface layer (0–10 cm) of the NT site (13.0 g/kg) was slightly higher than CT site (11.4 g/kg). However, this difference did not give a statistically significant result and, as a consequence, cannot explain the higher CO_2_ fluxes detected at NT site. Conversely, the total carbon content of the microbial biomass in the top soil mineral fraction (0–30 cm) was almost twice higher at the NT site than at CT site, resulting in 97.4 *μ*gC/g versus 55.8 *μ*gC/g, but once again this difference was not statistically significant.

Nevertheless, it is important to underline that there is a clear relation between increased microbial biomass and increased soil CO_2_ effluxes, as for example, shown in a recent work by Han et al. [45]. Moreover Sowerby et al. [46] clearly demonstrated that increased soil microbial biomass, rather than increased microbial activity, was responsible for the rise of soil CO_2_ effluxes observed in their experiment.

### 3.4. Influence of Weather

**Figure 5:**
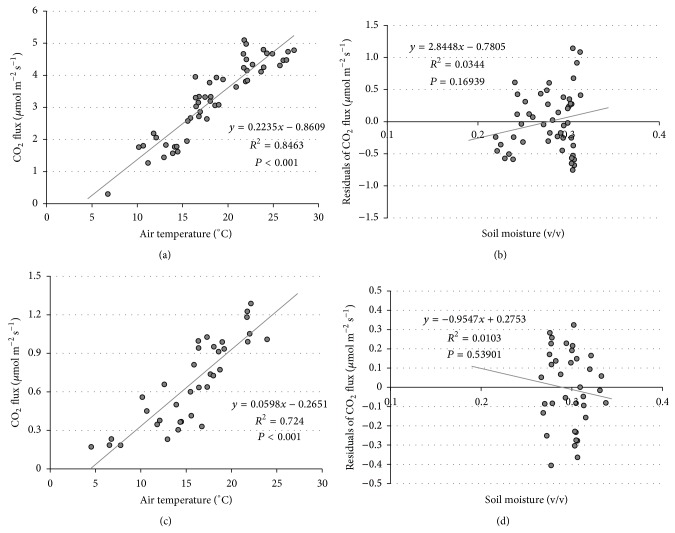
Linear regression between CO_2_ fluxes and air temperature and soil moisture in the NT site ((a), (b)) and CT site ((c), (d)). Points represent the daily averages calculated only in the days with full data capturing. The residuals of the CO_2_ fluxes which appear in the pictures (b) and (d) are the residuals of the linear regressions shown in the pictures (a) and (c). (CT = conventional tillage site; NT = no-tillage site.)

The main driver of the CO_2_ soil fluxes at both sites was temperature, as expected for the microbial metabolism. The correlation of the daily mean fluxes with the daily mean air temperatures, a good proxy of the soil temperature [47, 48], was significant at both sites (*R* ^2^ = 0.846  *P* < 0.001 and *R* ^2^ = 0.724  *P* < 0.001, at NT and CT resp.) even if the flux intensities were very different (Figures 5(a) and 5(c)).

On the contrary the CO_2_ flux decreased at both sites at increasing soil moisture (*R* ^2^ = 0.39, *P* < 0.001 at NT site, and *R* ^2^ = 0.17, *P* < 0.05 at CT site), following rain events.

However, once the effect of the temperature was removed from the data and the residuals were considered for the regression with the soil moisture, this decrease completely disappeared at both sites (*R* ^2^ = 0.034, *P* = 0.169 at NT and *R* ^2^ = 0.0103  *P* = 0.539 at CT site, Figures 5(b) and 5(d)).

Therefore, the decrease of CO_2_ observed with increasing soil water content is exclusively due to the reduction of air temperature that is linked to rain events, and which also affects the following days.

### 3.5. Daily Patterns of Soil CO_2_ Fluxes

In general, the diurnal fluctuations of the CO_2_ fluxes were weak. Nevertheless little differences of the average daily courses in the two periods were detected. In July-August the maximum flux intensity occurred in the central part of the day, while a slight flux minimum appeared in the same part of the day at September-October (Figure 4).

**Figure 6:**
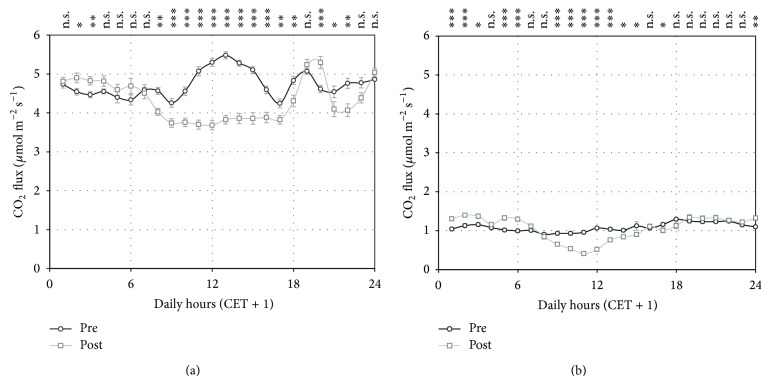
Comparison between the average daily courses of the CO_2_ fluxes of one week before (pre) and one week after (post) harvest at the NT site (a) and CT site (b). Vertical bars indicate the standard errors of the means. (CT = conventional tillage site; NT = no-tillage site.) The statistical significance of the differences between the flux daily courses of the two periods in both sites has been reported (for each hour) at the top of each graph (∗∗∗ for *P* < 0.001; ∗∗ for *P* < 0.01; ∗ for *P* < 0.05, and n.s. for *P* > 0.05).

The maximum flux observed at noon in July-August reflects the roots activity of the plants during the daylights. Indeed, this characteristic pattern disappeared in the postharvest period as shown in Figure 6, where the flux average daily courses recorded one week before and one week after the harvest is presented. The relative contribution of the root respiration to the diurnal CO_2_ fluxes can be estimated from the observation of the mean diurnal flux decrease (1 *μ*mol CO_2_ m^−2^ s^−1^ at the NT site and 0.8 *μ*mol CO_2_ m^−2^ s^−1^ at the CT site).

A relative minimum flux is clearly visible during the light hours after the harvest (Figure 5(b)) and lasts for the whole second period of measurements (Figure 4(b)).

### 3.6. Influence of Weedings

Weeds can play a possible role in determining the observed diurnal relative minimum of CO_2_ fluxes. In fact, the great increase of the sunlight at ground level, which followed the removal of the canopy with the harvest, could have stimulated the photosynthetic activity and growth of weeds and microalgae within the transparent Plexiglas chambers.

The role of weeds was confirmed with a comparison of the measurements taken three days before and three days after a chemical weeding treatment made at both sites in October (Figure 6). The diurnal flux minimum at the CT site (Figure 6(b)) completely disappeared afterthe herbicide treatment, while the relative flux depression was greatly reduced at the NT site after the same herbicide application (Figure 6(a)). Moreover, a slight reduction of the evening and night fluxes was observed at both sites.

### 3.7. Influence of Turbulence

The analysis of turbulence at ground level can help us to understand the residual diurnal flux minimum not explained by the weeds' photosynthesis at the NT site, after the herbicide treatment.

The direct exposure to the atmosphere of the two studied soils after the harvest caused an increase of the air turbulence at ground level, which rose from negligible values under the canopy to appreciable values after its removal (Figure 8), following dynamics that also other works reported [49–51].

**Figure 7:**
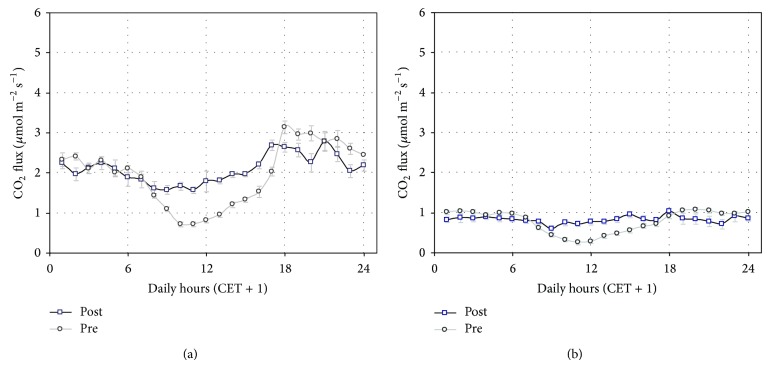
Effect of the application of a herbicide at the NT site (a) and at the CT site (b). Pretreatment and posttreatment hourly mean values are presented, based on measurements recorded 3 days before and 3 days after the herbicide treatment. Vertical bars indicate the standard errors of the means. (CT = conventional tillage site; NT = no-tillage site.)

**Figure 8:**
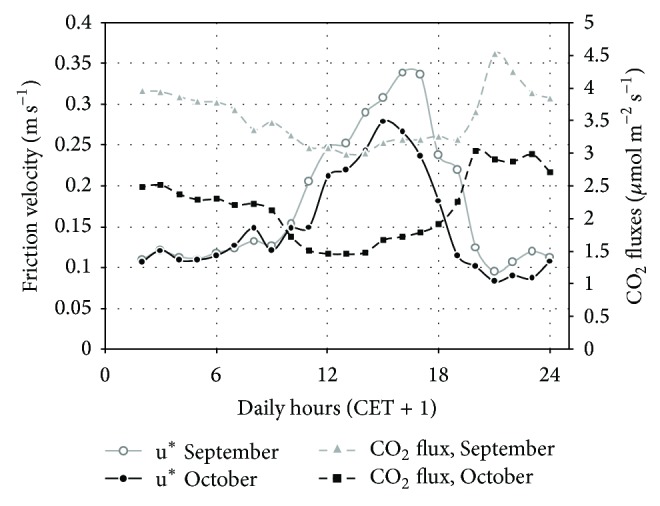
Atmospheric turbulence (mean daily pattern) and CO_2_ fluxes in the postharvest period (September-October) at the NT site. (NT = no-tillage site.)

Our results show that despite the CO_2_ fluxes were very different in their absolute values in the two postharvest months at NT site (Figure 6), the diurnal flux “depression” of about 1 *μ*mol m^−2^ s^−1^ shown by the daily courses of September and October was very similar. At the same time, the mean daily patterns of the air turbulence (*u* ^*^) in September and October were comparable during the daylight hours (Figure 7) and were identical during the night-time, thus suggesting a possible role of the air turbulence.

The effect of turbulence on the extraction of CO_2_ from the topsoil was well described by Takle et al. [52] who highlighted that the rapid fluctuations in the static pressure fields introduced by wind interactions with terrain may lead literally to pressure pumping of CO_2_ at the surface.

Moreover, in the presence of litter on the surface, like for example, in the NT site, the enhanced surface rugosity introduced by the litter can also contribute in increasing this process. In fact, Reicosky et al. [10] working with histosols with a bulk density between 0.23 and 0.27 Mg m^−3^ at the surface layer, a density which is barely different from the organic litter density observed at our site (0.14 ± 0.042 Mg m^−3^), has shown how wind speed can deplete the CO_2_ concentrations in highly porous organic soils.

It is likely that analogous processes occurred also at the NT site, where the litter layer could have acted as a buffer for the CO_2_ emitted from the underlying mineral topsoil. During the daylight hours, when the chambers were open, the increased turbulence could have caused a more intense removal of CO_2_ from the litter layer towards the atmosphere than during the night-time, thus leaving less exchangeable CO_2_ into the buffer. Hence, after the chamber's closure, the flux was lower during the daylight hours than during the night hours when the atmospheric turbulence was negligible (Figure 7). It is worth noticing that the particular SASSFLUX design (Figure 1) does not interfere with the wind action on the topsoil, because there are no emerging parts from the soil when the lid is open. It is likely therefore, that the relative minimum after the harvest is the result of the combination of two processes, photosynthesis/respiration of the weeds and air turbulence increase caused by litter.

## 4. Conclusion

In this paper CO_2_ emissions from the soil of two maize fields managed with conventional tillage and no-tillage techniques were compared at the time of maize harvest.

The NT site showed CO_2_ emissions up to 3.5 times higher than the CT site during the two months following the maize harvest. This result was related to the presence of a conspicuous organic litter on the NT site field's surface due to the accumulation of crop debris and residues in the last 10 years, and thus to an increased decomposition activity of the microbial community. However, the CO_2_ emission differences between the two fields slowly declined approaching the fall season, together with the flux intensity which was clearly driven by the air (and soil) temperature.

The spatial flux variability of the NT site field was higher than the CT site, reflecting a more irregular distribution of the organic matter on the soil surface and in the topsoil, which contrasts with the continuous redistribution of the organic matter along the soil profile in the CT site field.

However, results from this case-study do not allow us to get any conclusions on the environmental sustainability of the two different management practices, regarding their impacts on GHG emission and global warming, since they are focused only on one small aspect of the carbon budget of the studied agroecosystems.

Only a careful analysis of the carbon emissions during the whole life-cycle of the agroecosystem would allow a meaningful conclusion. And the simple inclusion in the budget of the CO_2_ emitted by the tractors employed for the mechanical treatments at the CT site (around 40 liters of fuel per hour) [53] would have radically changed any conclusion on the impacts on GHG emissions and global warning of the two different management practices.

The presented data can contribute to the development of high-time resolved modules for the CO_2_ exchange processes at the soil surface, to be included in more general models aimed at evaluating the total carbon budget of agroecosystems at regional scale.

## Figures and Tables

**Table 1:** Crop management practices in the two sites (CT = conventional tillage site, NT = no-tillage site). All dates are referred to year 2012.

	CT site	NT site
Plowing	(i) 21 May 30 cm depth tillage, mouldboard plough (ii) 22 May Harrow clods reduction	No-tillage

Species and cultivar	*Zea mays* cv Pioner P0222	*Zea mays* cv Decalb 6903

Sowing date	23 May	27 April

Sowing technique	Seedbed prepared using harrows/cultivators (1-2 passes)	Sod-seed

Seed population	7.8 seed m^−2^	8.6 seed m^−2^

Interrow spacing	70 cm	70 cm

Fertilizations	(i) 15 May Cattle manure: 100 m^3^ ha^−1^ C : N = 9.7, N: 200 kg ha^−1^ (ii) 23 May Perphosphate (46% of P), 30 Kg ha^−1^ (iii) 10 June Urea (46% of N), 150 kg ha^−1^	(i) 27 April Ammoniumsulphate N/S 21 : 24, 150 kg ha^−1^ Diammoniumphosphate N/P 18 : 46, 60 kg ha^−1^ (ii) 19 May 2012 Urea (46% of N), 175 kg ha^−1^

Irrigations	(i) 9 July 2012 Flooding (2000 m^3^ ha^−1^ per irrigation) (ii) 6 August 2012 Flooding (2000 m^3^ ha^−1^ per irrigation)	(i) 5 July 2012 Flooding (2000 m^3^ ha^−1^ per irrigation) (ii) 27 July 2012 Flooding (2000 m^3^ ha^−1^ per irrigation)

Open field weeding	(i) 21 June 2012 Du Pont “Codacide” 1.0 L ha^−1^ Syngenta “Callisto” 0.5 L ha^−1^ Du Pont “Titus ultra” 50 g ha^−1^ (ii) 2 October 2012 Dow “Hopper” (glyphosate), 3.5 L ha^−1^	(i) 27 April 2012 Syngenta “Force” 12 kg ha^−1^ (ii) 28 April 2012 Bayer “Merlin Gold” 1.2 L ha^−1^ Dow “Hopper” (glyphosate) 3 L ha^−1^ (iii) 26 May 2012 Du Pont “Codacide” 1.3 L ha^−1^ Syngenta “Callisto” 0.5 L ha^−1^ Du Pont “Titus ultra” 42 g ha^−1^ (iv) 8 October 2012 Dow “Hopper” (glyphosate), 3 L ha^−1^

Weedings within SASSFLUX	(i) 16 July Dow “Hopper” (glyphosate) 3 L ha^−1^ (ii) 28 September Dow “Hopper” (glyphosate) 3 L ha^−1^ (iii) 24 October Dow “Hopper” (glyphosate) 3 L ha^−1^	(i) 16 July Dow “Hopper” (glyphosate) 3 L ha^−1^ (ii) 28 September Dow “Hopper” (glyphosate) 3 L ha^−1^ (iii) 24 October 2012 Dow “Hopper” (glyphosate) 3 L ha^−1^

Harvest date	2 September	22 August

Harvest technique	One combined harvester (thresher and chopper machine for silage) Two tractors with wagon for fine	One combined harvester (thresher and chopper machine) Two tractors with wagon for fine

**Table 2:** Soil and litter organic carbon content in the two experimental sites. In both sites, soil type was Luvisol, sandy loam, subacid, and well drained. CT = conventional tillage site; NT = no-tillage site.

	CT site	NT site
Soil type	Luvisol, sandy loam, sub acid, and well drained	Luvisol, sandy loam, sub acid, and well drained
Bulk density of the soil	1.57 ± 0.10 g cm^−3^	1.57 ± 0.07 g cm^−3^
Organic carbon of the topsoil (0–30 cm)	10.3 ± 1.8 g kg^−1^	9.5 ± 0.2 gC kg^−1^
Carbon stock in the topsoil	48.7 ± 6.3 t ha^−1^	44.8 ± 5.6 t ha^−1^
Organic carbon in the microbial biomass	55.8 ± 16.2 *μ*gC g^−1^	97.4 ± 41.5 *μ*gC g^−1^
Litter mass	No litter	1.70 ± 0.4 kg m^−2^
Organic carbon in the litter	No litter	237.9 ± 90 gC kg^−1^
Carbon stock in the litter	No litter	4.2 ± 2.0 t ha^−1^

**Table 3:** Weather conditions measured at the NT site during the data measuring campaign.

Month	Mean air temp. (°C)	Average of maximum air temp. (°C)	Average of minimum air temp. (°C)	Rain (mm)	Average soil water content (v/v)	Average soil heat flux^*^ (W/m^2^)
July	22.73	29.20	16.59	12.6	0.26	−0.69
August	24.73	31.51	17.82	24.6	0.25	0.11
September	18.57	24.15	14.30	74.2	0.28	−6.75
October	12.45	17.12	9.73	87.6	0.30	−8.59

^*^Negative values indicate losses of thermal energy toward the atmosphere.
